# Real-world ANASTASE study of atezolizumab+nab-paclitaxel as first-line treatment of PD-L1-positive metastatic triple-negative breast cancer

**DOI:** 10.1038/s41523-023-00579-2

**Published:** 2023-09-08

**Authors:** Alessandra Fabi, Luisa Carbognin, Andrea Botticelli, Ida Paris, Paola Fuso, Maria Cristina Savastano, Nicla La Verde, Carla Strina, Rebecca Pedersini, Stefania Guarino, Giuseppe Curigliano, Carmen Criscitiello, Mimma Raffaele, Alessandra Beano, Antonio Franco, Maria Rosaria Valerio, Francesco Verderame, Andrea Fontana, Eva Regina Haspinger, Alessia Caldara, Alba Di Leone, Giampaolo Tortora, Diana Giannarelli, Giovanni Scambia

**Affiliations:** 1 Precision Medicine Unit in Senology, Fondazione Policlinico Universitario A. Gemelli IRCCS, Rome, Italy; 2 Division of Gynecology Oncology, Department of Woman and Child Health and Public Health, Fondazione Policlinico Universitario A. Gemelli IRCCS, Rome, Italy; 3 Medical Oncology Unit, La Sapienza, University of Rome, Policlinico Umberto I, Rome, Italy; 4 Medical Oncology Unit, A.O.U. San Giovanni di Dio e Ruggi D’Aragona, Salerno, Italy; 5 Medical Oncology Unit, ASST Fatebenefratelli Sacco PO Luigi Sacco - Polo Universitario, Milan, Italy; 6 Medical Oncology Unit Azienda Socio-Sanitaria Territoriale Cremona, Cremona, Italy; 7 Medical Oncology Unit ASST Spedali Civili, Brescia, Italy; 8 Medical Oncology Unit Santa Maria della Misericordia Hospital, Urbino, Italy; 9 Department of Oncology and Hemato-Oncology, University of Milan, Milan, Italy; 10 Division of Early Drug Development, European Institute of Oncology, IRCCS, Milan, Italy; 11 Presidio Cassia Sant’andrea, Dipartimento Oncologico, Asl Roma1, Rome, Italy; 12 Department of Medical Oncology1, Città della Salute e della Scienza Hospital, Turin, Italy; 13 Breast Unit, Department of Women, Children and Public Health Sciences, Fondazione Policlinico Universitario Agostino Gemelli IRCCS, Rome, Italy; 14 Medical Oncology, Policlinico Universitario P. Giaccone, Palermo, Italy; 15 Medical Oncology, AO Riuniti Villa Sofia, Cervello, Palermo, Italy; 16 Medical Oncology Unit 2, Azienda Ospedaliero-Universitaria Pisana, Pisa, Italy; 17 Azienda Sanitaria dell’Alto Adige – Ospedale di Merano, Merano, Italy; 18 Santa Chiara Hospital, Trento, Italy; 19 Medical Oncology, Fondazione Policlinico Universitario A. Gemelli IRCCS, Rome, Italy; 20 Medical Oncology, Department of Translational Medicine and Surgery, Università Cattolica del Sacro Cuore, Rome, Italy; 21 Epidemiology and Biostatistics Facility, Fondazione Policlinico Universitario A. Gemelli IRCCS, Roma, Italy; 22 Università Cattolica del Sacro Cuore, Rome, Italy

**Keywords:** Breast cancer, Breast cancer

## Abstract

The combination of atezolizumab and nab-paclitaxel is recommended in the EU as first-line treatment for PD-L1-positive metastatic triple-negative breast cancer (mTNBC), based on the results of phase III IMpassion130 trial. However, ‘real-world’ data on this combination are limited. The ANASTASE study (NCT05609903) collected data on atezolizumab plus nab-paclitaxel in PD-L1-positive mTNBC patients enrolled in the Italian Compassionate Use Program. A retrospective analysis was conducted in 29 Italian oncology centers among patients who completed at least one cycle of treatment. Data from 52 patients were gathered. Among them, 21.1% presented de novo stage IV; 78.8% previously received (neo)adjuvant treatment; 55.8% patients had only one site of metastasis; median number of treatment cycles was five (IQR: 3–8); objective response rate was 42.3% (95% CI: 28.9–55.7%). The median time-to-treatment discontinuation was 5 months (95% CI: 2.8–7.1); clinical benefit at 12 months was 45.8%. The median duration of response was 12.7 months (95% CI: 4.1–21.4). At a median follow-up of 20 months, the median progression-free survival was 6.3 months (95% CI: 3.9–8.7) and the median time to next treatment or death was 8.1 months (95% CI: 5.5–10.7). At 12 months and 24 months, the overall survival rates were 66.3% and 49.1%, respectively. The most common immune-related adverse events included rash (23.1%), hepatitis (11.5%), thyroiditis (11.5%) and pneumonia (9.6%). Within the ANASTASE study, patients with PD-L1-positive mTNBC treated with first-line atezolizumab plus nab-paclitaxel achieved PFS and ORR similar to those reported in the IMpassion130 study, with no unexpected adverse events.

## Introduction

Triple-negative breast cancer (TNBC) represents 15–20% of all breast cancers (BCs); it is characterized by the lack of expression of estrogen receptor (ER), progesterone receptor (PR), and the absence of *HER2* gene amplification^1,2^. Compared with other BC subtypes, TNBCs are often histologically high-grade tumors characterized by strong invasiveness and higher rates of relapse and mortality^3,4^. Unlike other BC subtypes that harbor therapeutic targets, such as ER or HER2, in the metastatic TNBC (mTNBC) subtype, systemic chemotherapy remains the standard of care. However, new targeted therapies, such as PARP inhibitors, immune checkpoint inhibitors (ICIs), and antibody−drug conjugates (e.g., sacituzumab−govitecan or trastuzumab deruxtecan in *HER2* low BC) are now available^5–8^.

However, patients with metastatic mTNBC have a median overall survival (OS) of less than 18 months with standard chemotherapy, making mTNBC a clinical challenge to treat, highlighting the need for more effective targeted therapies or combinations^6,9–12^.

TNBC is more likely to have increased expression of the PD-L1 in the tumor microenvironment, making it an ideal candidate for targeted therapy with ICIs^6,12–14^. Initial trials with ICIs in BC were conducted as monotherapy, but because of the limited benefit observed, research shifted to testing combinatorial approaches^5^. In particular, the combination of atezolizumab and nab-paclitaxel, within the randomized phase III IMpassion130 study, demonstrated a benefit for patients with mTNBC and PD-L1-positive by Ventana SP142 assay; in particular, the study met its co-primary progression-free survival (PFS) endpoint in the intention-to-treat population and patients with PD-L1-positive in ≥1% immune cells (IC+). Improved activity of a such combination was observed only in patients whose tumors were PD-L1-positive, and in these patients, a clinically meaningful OS improvement was also observed^15^. On these bases, the combination of atezolizumab and nab-paclitaxel has been approved in Europe as a first-line treatment option for PD-L1-positive unresectable locally advanced or mTNBC^16^, thus setting a new standard of care.

However, ‘real-world’ data on both the efficacy and safety of this combination are limited. To fill this gap, we designed the multicentre, real-world ANASTASE study, which aimed to evaluate the therapeutic effectiveness and safety of atezolizumab plus nab-paclitaxel in a cohort of Italian patients with PD_l1-positive metastatic or unresectable locally advanced TNBC enrolled in the Compassionate Use Program (CUP).

## Results

### Patient characteristics

Data from 52 patients were gathered. The clinical features of the study population are summarized in Table 1. The median age at the initial diagnosis was 52 years (IQR: 45–63 years), and 65.4% (*n* = 34) of patients were in postmenopause. At diagnosis, 11 patients (21.1%) presented de novo stage IV, and *BRCA* mutation was identified in eight patients (15.4%) among 37. Of the 41 (78.8%) non-metastatic patients at their first diagnosis, most of them had previously received neoadjuvant (*n* = 15, 36.6%) or adjuvant (*n* = 18, 43.9%) or both (*n* = 7, 17.1%) treatments, including a taxane-based and anthracycline-based chemotherapy regimen in 65.4% and 69.2% of cases, respectively; the median disease free-interval was 20 months (IQR: 13–50). Concerning the number and the site of metastases at the diagnosis of metastatic disease, 29 (55.8%) patients had only one site of metastasis, six (11.5%) had three or more metastatic sites, 31 (59.6%) had visceral metastases, and two (3.8%) had brain metastases.

**Table 1:** Clinical features of the study population (*n* = 52).

Variable	Patients, *n* (%)
Menopausal stage at diagnosis:	
• Premenopausal	17 (32.7)
• Postmenopausal	34 (65.4)
• Unknown	1 (1.9)
ECOG PS at the time of advanced disease diagnosis:	
• 0	40 (77)
• 1	8 (15.4)
• 2	1 (1.9)
• Unknown	3 (5.7)
*BRCA1*/*2* status:	
• Positive	8 (15.4)
• Negative	29 (55.8)
• Unknown	15 (28.8)
Disease stage at initial diagnosis:	
• 1	4 (7.7)
• 2	15 (28.8)
• 3	22 (42.3)
• 4	11 (21.1)
Surgery of the primary^a^	44 (84.6)
Previous systemic therapy	
Total patients	41 (78.8)
• Only neoadjuvant chemotherapy	15 (36.6)
• Only adjuvant chemotherapy	18 (43.9)
• Both neoadjuvant and adjuvant chemotherap*y*	7 (17.1)
• Nihil	1 (2.4)
Previous neo/adjuvant chemotherapy regimen:	
• Anthracyclines	36 (69.2)
• Taxane	34 (65.4)
• Carboplatin	8 (15.4)
Number of metastatic sites:	
• 1	29 (55.8)
• 2	17 (32.7)
• ≥3	6 (11.5)
Site of metastasis at the time of advanced disease diagnosis:	
• Liver	7 (13.5)
• Lung	25 (48.1)
• Bone	9 (17.3)
• Soft tissue	34 (65.4)
• Brain	2 (3.8)
Dominant site of metastasis:	
• Liver	7 (13.5)
• Lung	24 (46.2)
• Bone	5 (9.6)
• Soft tissue	16 (30.8)

*ECOG* Eastern Cooperative Oncology Group, *PS* Performance Status.

^a^Three patients with de novo metastatic disease had palliative surgery of primary breast cancer.

### Exposure to drugs

Treatment exposure and features are summarized in Table 2. The median number of nab-paclitaxel and atezolizumab cycles was five (IQR: 2–6 cycles) and six (IQR: 3–8), respectively. Overall, 50 (96.2%) patients discontinued nab-paclitaxel, and 45 (86.5%) patients discontinued atezolizumab, mainly due progressing disease in 66% and 80% of cases, respectively. Treatment discontinuation rates due to AEs were 14% and 13.3% for nab-paclitaxel and atezolizumab, respectively. Regarding maintenance, eight patients (15.4%) received atezolizumab monotherapy for a median number of six cycles (IQR: 3–8). No differences in terms of baseline characteristics were reported between patients receiving atezolizumab maintenance therapy compared with no maintenance treatment (Supplementary Table 1). A trend for patients with single metastatic site in favour of receiving atezolizumab maintenance compared to multiple metastatic sites was reported (Supplementary Table 1). Finally, eight patients (15.4%) were still on treatment at the analysis time.

**Table 2:** Treatment characteristics of the study population (*n* = 52).

Variable	Patients, *n* (%)
Nab-paclitaxel discontinuation:	
• Yes	50 (96.2)
• No	2 (3.8)
Reason for Nab-paclitaxel discontinuation:	
• Toxicity	7 (14.0)
• Disease progression	33 (66.0)
• Physician decision	8 (16.0)
• Patient decision	2 (4.0)
Atezolizumab discontinuation:	
• Yes	45 (86.5)
• No	7 (13.5)
Reason for atezolizumab discontinuation:	
• Toxicity	6 (13.3)
• Disease progression	36 (80.0)
• Physician decision	1 (2.2)
• Patient decision	2 (4.4)
Atezolizumab maintenance:	
• Yes	8 (15.4)
• No	44 (84.6)

### Activity results

All the patients were evaluable for time to treatment discontinuation (TTD), while a total of 48 patients out of 52 were evaluable for response (two patients were not evaluable due to missing data, and two patients did not complete the first treatment cycle due to AEs). Response outcomes are reported in Table 3. The objective response rate (ORR) was obtained in 22 patients (42.3%; 95% CI: 28.9–55.7%), including 5.8% (*n* = 3) of complete response and 36.5% (*n* = 19) of partial response. A total of 16 (30.8%) patients had progressive disease. Stable disease was reported in 10 (19.2%) patients.

**Table 3:** Response outcomes (*n* = 52).

Outcomes	Patients
Overall objective response, *n* (%) [95% CI]	22 (42.3) [28.9–55.7]
Complete response, *n* (%)	*3* (5.8)
Partial response, *n* (%)	*19* (36.5)
Stable disease, *n* (%)	10 (19.2)
Progressive disease, *n* (%)	16 (30.8)
Patients who had missing data or could not be evaluated, *n* (%)	*4* (7.7)
Median duration of response (95% CI); months	12.7 [4.1–21.4]
Median cycle to best response (95% CI); months	3.0 (1–7)

**Fig. 1:**
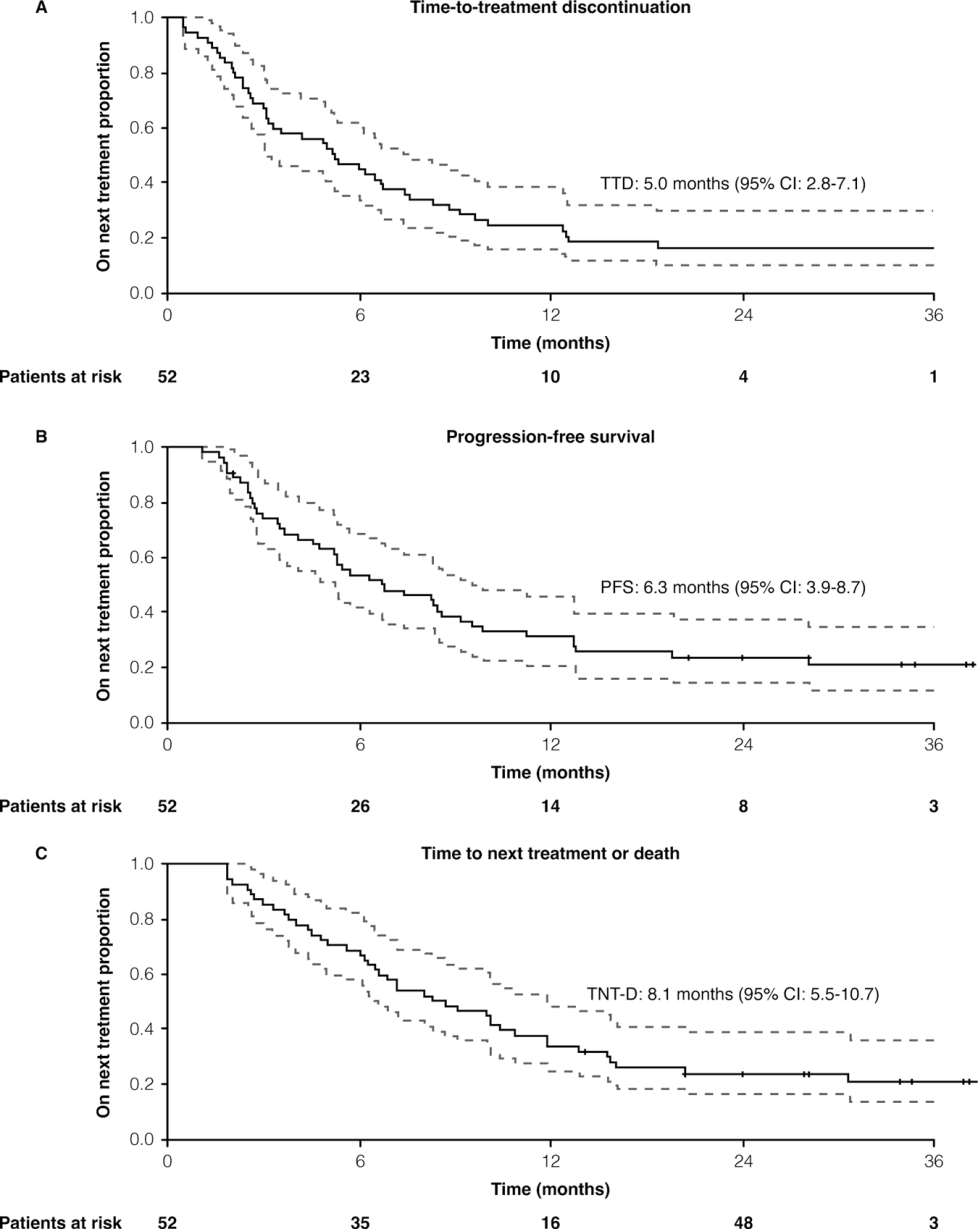
Overall survival of study population. **A** Kaplan–Meier curve of the time to treatment discontinuation (TTD) of anastase study population. Median TTD was 5.0 months (95%CI: 2.8–7.1). **B** Kaplan–Meier curve of the progression free survival (PFS) of anastase study population. Median PFS was 6.3 months (95%CI: 3.9–8.7). **C** Kaplan–Meier curve of the time to next treatment or death (TNT-D) of anastase study population: Median TNT-D was 8.1 months (95%CI: 5.5–10.7).

The median TTD was 5 months (95% CI: 2.8–7.1 months; Fig. 1A) for the overall population. There was no difference in terms of median TTD between patients treated with or without previous anthracyclines regimens in early disease (5 months [95% CI: 2.5–7.5 months) and 4.9 months (95% CI: 0–10.9 months). The clinical benefit at 6 and 12 months was 54.2% and 45.8%, respectively.

Concerning the DoR, the median time was 12.7 months (95% CI: 4.1–21.4 months) with a median cycle to best response of 3 months (95% CI: 1–7 months) (Table 3).

### Efficacy results

At a median follow-up of 20 months (IQR: 16–24 months), the median PFS was 6.3 months (95% CI: 3.9–8.7 months) (Fig. 1B). When analyzing the TNT-D, the median time was 8.1 months (95% CI: 5.5–10.7 months) (Fig. 1C). At 12 months and 24 months, the OS rates were 66.3% and 49.1% (Supplementary Fig. 1), respectively. No statistically significant difference was reported in terms of ORR, PFS, and TNT-D according to different subgroups such as stage at diagnosis, number and type of metastatic sites, *BRCA1*–*2* mutational status, disease-free interval, ECOG PS, *HER2* status (0 vs low) and previous treatment (Supplementary Tables 2–4).

### Outcome after atezolizumab plus nab-paclitaxel progression

Among the 43 patients with progressive disease after atezolizumab plus nab-paclitaxel, thirty-one patients (59.6%) received second-line therapy; among them, eight (25.8%) and seven (22.6%) patients received regimens including carboplatin and capecitabine, respectively. Median PFS on second-line therapy was 6.9 months (IQR: 3.1–10.7 months). The second-line treatments are summarized in Table 4.

**Table 4:** Second-line therapy after nab-paclitaxel and atezolizumab.

Treatment	Patients, *n* (%)
Patients receiving a second-line therapy:	31 (59.6)
• Carboplatin-based regimen	8 (25.8)
• Capecitabine	7 (22.6)
• Anthracycline-based regimen	5 (16.1)
• Eribulin	5 (16.1)
• Olaparib	3 (9.7)
• Docetaxel	1 (3.2)
• CMF	1 (3.2)
• Ipatasertib	1 (3.2)

### Safety

All 52 patients were available for safety. The most common AEs of any grade included neutropenia (57.7%), anemia (53.8%), lymphocytopenia (46.2%), asthenia (46.2%), liver toxicity (40.4%), nausea and vomiting (32.7%) (Table 5).

**Table 5:** Adverse Events (all adverse events suggestive of potential immune-related etiology were listed).

Event	Patients, *n* (%)			Median cycle of onset
	Any grade	G1−2	G3−4	
Neutropenia	30 (57.7)	26 (50.0)	4 (7.7)	2
Lymphocytopenia	24 (46.2)	18 (34.6)	6 (11.5)	2
Anemia	28 (53.8)	27 (51.9)	1 (1.9)	2
Thrombocytopenia	9 (17.3)	9 (17.3)	0	4
Asthenia	24 (46.2)	23 (44.3)	1 (1.9)	3
Liver toxicity^a^	21 (40.4)	20 (38.5)	1 (1.9)	2
Nausea	17 (32.7)	16 (30.8)	1 (1.9)	2
Vomiting	17 (32.7)	17 (32.7)	0	2
Rash cutaneous	12 (23.1)	12 (23.1)	0	2
Diarrhea	10 (19.2)	10 (19.2)	0	3
Peripheral neuropathy	10 (19.2)	8 (15.4)	2 (3.8)	2
Stomatitis	10 (19.2)	10 (19.2)	0	2
Hepatitis	6 (11.5)	5 (9.6)	1 (1.9)	3
Thyroiditis	6 (11.5)	6 (11.5)	0	3
Pneumonia	5 (9.6)	5 (9.6)	0	2
Fever	5 (9.6)	5 (9.6)	0	3
Onychopathy	2 (3.8)	2 (3.8)	0	3

^a^Transaminase increasing.

The most common potential immune-related AEs of any grade included: rash (23.1%), thyroiditis (11.5%), hepatitis (11.5%) and pneumonia (9.6%). Grade 3 or 4 of these events occurred only in one patient with hepatitis (Table 5).

## Discussion

Our paper reports data on atezolizumab plus nab-paclitaxel in PD-L1-positive mTNBC patients enrolled in the Italian Compassionate Use Program within the ANASTASE study. Tumor heterogeneity and the long-standing paucity of effective therapies other than chemotherapy have contributed to TNBC being the subtype with the least favorable outcomes^3,4,17^. In recent years, advances in -omics technologies have shed light on the relevance of the TNBC microenvironment heterogeneity, unveiling a close dynamic relationship with cancer cell features^17^. In particular, TNBC resulted as the most immunogenic BC subtype, with higher PD-L1 expression levels and more tumor-infiltrating lymphocytes^18–20^. These assumptions have led to the development of novel targeted agents, including ICIs, revolutionizing the therapeutic landscape and providing new therapeutic opportunities^6^.

Patients with PD-L1-positive TNBC are the most likely to benefit from ICIs^21^. Notably, the IMpassion130 trial established the utility of adding atezolizumab to nab-paclitaxel as the first-line treatment for mTNBC, with most of the clinical benefit realized in the PD-L1-positive subgroup^15,22^. On this basis, the results of the IMpassion130 trial led to the accelerated approval of atezolizumab in combination with nab-paclitaxel for patients with unresectable locally advanced tumors or mTNBC whose tumors express PD-L1. This represents a major breakthrough in BC treatment because of the novelty of immunotherapy in BC and the improved outcome benefit compared with chemotherapy alone^16^. Despite atezolizumab indication has been withdrawn from the USA given negative findings in the IMpassion 131 trial, the combination of atezolizumab and nab-paclitaxel is currently authorized in Europe as a first-line treatment for PD-L1-positive mTNBC^16^.

Within our cohort, 19% of patients were de novo metastatic. Compared with the IMpassion130 study, a higher percentage of patients (65% ANASTASE vs 51% IMpassion130 study) had adjuvant therapy with taxanes; otherwise, the disease burden was similar, with most patients reporting between zero and three metastatic sites^15^. We observed an ORR of 42.3%, which is a lower rate than in the IMpassion130 study (58.9%) (Supplementary Table 5)^15^. TTD, describing the period from the treatment initiation to discontinuation or death, has been proposed as a potential effectiveness endpoint for real-world studies where imaging assessment is less structured and standardized^23^. In our study, the median TTD was 5 months (95%, CI: 2.8–7.1), slightly shorter than the median PFS (6.3 months; 95% CI: 3.9–8.7 months). These data align with recent findings from a patient-level correlation analysis comprising 18 clinical trials in advanced non-small-cell lung cancer^23^. This analysis showed that with ICI therapy, the median PFS is slightly longer than the median TTD, with both early and late TTD cases^23^. This suggests that, in some cases, patients terminated the ICI treatment because of immune-mediated AEs and continued to have sustained benefits after treatment discontinuation.

Comparable results in terms of PFS (6.3 vs 7.5 months) were observed with the IMpassion130 trial (Supplementary Table 5)^15^. Otherwise, a longer median DoR was observed in our study, compared with the IMpassion130 (12.7 months vs 8.5) (Supplementary Table 5)^15^. The selection of the most treatment-responsive patients remains unresolved to date, and the definition of biomarkers to optimize both patient and treatment selection is still an unmet need^24^. As mentioned above, a recent sub-study from the IMpassion130 trial reported that a clinical benefit was observed only in PD-L1 IC+ patients. However, the combination treatment was more efficacious in patients with richer tumor immune microenvironments^22^. Therefore, our finding supports the observation that patients sensitive to combined treatment have a prolonged benefit, highlighting the importance of patient selection to improve the clinical benefit of treatments.

The analysis of TNT-D represents interesting information in a population with poor prognosis after progression from first-line treatment, such as triple-negative patients. In particular, an attractive characteristic of TNT-D is its ability to capture the treatment-free interval from the end of index therapy to the date of initiation of a subsequent line of treatment or death^25^. Within the ANASTASE study, we analyze the TNT-D in a TNBC population treated with ICI for the first time, reporting a median of 8.1 months (95% CI: 5.5–10.7 months). These data further support the benefit of ICI therapy through a prolonged treatment effect in clinical practice. At 12 months and 24 months, the OS rates were 66.3% and 49.1%, respectively.

In 2020, the FDA granted accelerated approval of pembrolizumab, another PD-1 inhibitor, in combination with chemotherapy for locally recurrent unresectable and metastatic PD-L1-positive TNBC, based on the results of phase III Keynote-355 trial^26^. In particular, this trial showed a statistically significant PFS benefit with the addition of pembrolizumab to chemotherapy in patients with a combined positive score ≥10. This benefit was more pronounced if pembrolizumab was associated with a taxane regimen^26^. However, PD-L1 positivity was defined by two different tests in the IMpassion130 and Keynote-355 trials: Ventana SP142 and Dako 22C3 assays, respectively. Utilizing the Dako 22C3 assay to select PD-L-1-positive tumors on the biobank from IMpassion130, considering a combined positive score ≥10, the median OS was 22 months with atezolizumab versus 18.7 months (hazard ratio, HR: 0.77), compared with 25 months with atezolizumab versus 18 months (HR: 0.71) via SP142 assay^27^. For these reasons, when using the Ventana SP142 assay, atezolizumab and nab-paclitaxel are recommended as standard of care for patients with mTNBC whose tumors have a ≥1% PD-L1-positive score in Europe^28^. In particular, this combination represents the only first-line treatment registered and reimbursed in Italy for TNBC patients with PD-L1 ≥ 1% (Ventana SP142 assay). Consistent with observations from other atezolizumab–chemotherapy combination trials, no unexpected AEs were observed^15,29,30^. Of note, previous literature evidence has shown that the unique spectrum of AEs associated with ICIs requires supplementary monitoring and treatment practices more than those required for chemotherapy^31^. In our cohort, no high incidence of severe toxicity was reported; one patient reported severe hepatitis leading to treatment discontinuation. Only two cases of grade 2 pneumonia were observed: one achieved after the first cycle, treated with antibiotic and steroid therapy, and recovered in 1 month without further treatment interruption; the other patient presented this AE after five cycles of therapy and temporarily stopped treatment until there was an improvement to grade 1.

This study presents some limitations, such as the relatively small sample size and the retrospective and real-world nature. However, the compassionate use programs, such as the ANASTASE study, are characterized by some relevant values: offer a controlled system of access to new experimental drugs before the commercialization and completely outside of clinical trial, commonly to patients with life‐threatening diseases and with no therapeutic options or in case of highly active drugs also in early therapeutic approach^32^. Furthermore, the compassionate use can also permit to clinicians to have more confidence with new drugs or regimens in terms of toxicity management. Since the ANASTASE study represented the first experience for clinicians of the compassionate use of immunotherapy in TNBC, toxicity management was not based on previous clinical experiences. Moreover, our study provides real-world evidence, which is of increasing interest to support clinical decision-making, as it could fill gaps by supporting the generation of evidence for subsequent indications, optimal dosing, and studying special populations, as well as providing information on the management of toxicities in the manner and scope of the real-world clinical practice^23^. On this regard, a series of previous compassionate use experience has been reported in the context of BC^32–34^. We would like also to specify that although the CUP excluded all patients with TFI <12 months, in relation to the inclusion and exclusion criteria of the IMpassion130, it was considered important to also include patients with TFI <12 months in the ANASTASE study to gather preliminary information on the activity of combination in the real world in this subsetting of patients. The combination of atezolizumab plus chemotherapy in the first-line mTNBC PD-L1-positive patients with DFI <12 months is also currently being studied in the prospective phase III study Impassion 132 (NCT03371017).

Recently, the Keynote-522 study showed that neoadjuvant pembrolizumab plus chemotherapy, followed by adjuvant pembrolizumab after surgery, resulted in significantly longer event-free survival than neoadjuvant chemotherapy alone in early TNBC^35^. To date, the combination of pembrolizumab and chemotherapy is the standard of care for high-risk, early-stage TNBC, regardless of PD-L1 status. However, the neoadjuvant use of pembrolizumab and chemotherapy results, upon disease progression, in a degree of uncertainty regarding the use of ICI in the first line, due to the lack of data on patients progressing after neoadjuvant therapy with ICI and treated in the first-line setting with this regime. Consequently, new data from this patient setting are awaited.

Our findings suggest that PD-L1- positive mTNBC patients treated with first-line atezolizumab plus nab-paclitaxel substantially achieved, in a ‘real-word’ context, a similar PFS to that reported in the IMpassion130 study, despite a lower response rate. The combination of atezolizumab and nab-paclitaxel appeared safe, with no unexpected AEs. A longer median DoR was observed in our study, compared with the IMpassion130, highlighting the importance of patient selection to improve the clinical benefit of the treatment. In addition, we provided the first evaluation of TTD and TNT-D, suggesting that ICI may find benefits even after treatment discontinuation in clinical practice.

## Patients and methods

### Study design and setting

ANASTASE study was a retrospective, multicenter, observational trial conducted in 29 Italian oncology centers to evaluate the therapeutic effectiveness and safety of the combination of atezolizumab plus nab-paclitaxel in a real-life context. The study involved PD-L1-positive metastatic or locally advanced TNBC adult patients who completed at least the first cycle of atezolizumab and nab-paclitaxel treatment within the CUP AL41712 (active from November 2019 to August 2020). No prior chemotherapy, experimental or targeted systemic therapy for mTNBC was allowed. Prior chemotherapy (including anthracyclines and taxanes) in the neoadjuvant or adjuvant setting was allowed if treatment was completed ≥12 months prior to the start of atezolizumab plus nab-paclitaxel treatment. Atezolizumab plus nab-paclitaxel were administered as follows: atezolizumab 840 mg intravenous (iv), on days 1 and 15 associated with nab-paclitaxel 100 mg/m^2^ iv on days 1, 8, and 15, every 28 days, at the same dose and frequency as the IMpassion130 study^15^. The treatment continued until disease progression, unacceptable toxicity, or a patient’s or physician’s request to discontinue. Grade 3 or 4 toxic effects were managed by dose modifications. Concomitant treatments that did not interfere with both drugs, including the use of bisphosphonates, were admitted. The study was conducted in accordance with the ethical standards of the Declaration of Helsinki and its subsequent amendments and within the protocol approved by the ethics committee of Fondazione Policlinico Universitario A Gemelli IRCCS of Rome (Italy; protocol number 25493/22). All participants provided written informed consent to the use of medical records for research purposes. ClinicalTrials.gov identifier: NCT05609903.

### Study measures

The primary objectives were to describe the overall population, including patients who completed at least the first cycle of treatment, estimate the time-to-treatment discontinuation (TTD, defined as the time from initiation of therapy to discontinuation of treatment for any reason), the ORR, using RECIST v1.1, the assessment of clinical benefit at 6 and 12 months, and assess the safety-evaluable population (including all patients who received at least one cycle of the study drug).

The secondary objectives were to estimate the duration of response (DoR) among patients with an objective response, defined according to the clinical practice, the median PFS (time to the start of treatment to the first progression of disease), time to next treatment or death (TNT-D, intended as the time to the start of the therapy to the date of next subsequent systemic treatment initiation or death, whichever occurs first), and OS rate, as well as to describe second-line therapy after atezolizumab plus nab-paclitaxel progression. The incidence of adverse events (AEs) suggestive of potential immune-related etiology was also assessed.

### Data retrieval

Demographics, medical history, BC history, and tumor biology were collected within the CUP, active in Italy, from November 2019 to August 2020. The primary data source was the medical record of the patient. The expression of conventional biological factors, such as *ER* and *PR*, *HER2* status, and Ki-67 proliferation index, was obtained from pathology reports. Triple-negative subtype was defined as ER- and PR-negative, and *HER2*-negative. The ER-negative and PR-negative status was defined if the percentage of positive nuclei by immunohistochemical (IHC) method was <1%. The *HER2*-negative status was defined if a 0, 1+ or 2 + IHC score was found with non-amplified in situ hybridization, according to the ASCO-CAP 2018 guidelines^36^. PD-L1-positive tumor status was defined as PD-L1 expression ≥1% on tumor-infiltrating immune cells as a percentage per tumor area, assessed by the Ventana PD-L1 (SP142) assay based on the status of the primary tumor and/or the biopsy of metastatic disease before starting treatment. Samples should have been evaluated by a qualified laboratory, and different assays are not acceptable. Response to atezolizumab plus nab-paclitaxel was evaluated according to the Response Evaluation Criteria in Solid Tumors (RECIST) criteria^37^. Toxicity was evaluated by the National Cancer Institute Common Terminology Criteria for Adverse Events (version 4.02) and according to Italian laws^38–40^. All AEs and serious AEs considered related to atezolizumab, and nab-paclitaxel were documented in the source data.

### Statistical analysis

Data were summarized using absolute counts and percentages when considering categorical variables and median values, and interquartile range (IQR) when referring to quantitative items. Differences in ORR between subgroups were assessed using the chi-square test. Survival times were estimated by the Kaplan-Meier method, and median values were reported with their 95% CIs. Differences between the curves were evaluated with the log-rank test. The sample size was not determined previously, as this analysis was performed on patients participating in the Expanded Access Program for atezolizumab according to their clinician’s decision. A subgroup analysis was planned; *p*-values are to be considered in an exploratory approach. IBM SPSS Statistics for Windows v.28.0 (Armonk, NY) was used for analysis.

### Supplementary information

Supplementary Materials

reporting-summary

## Data Availability

The datasets generated during and/or analyzed during the current study are available from the corresponding author upon reasonable request.
