# Author Correction: Real-world ANASTASE study of atezolizumab+nab-paclitaxel as first-line treatment of PD-L1-positive metastatic triple-negative breast cancer

**DOI:** 10.1038/s41523-024-00619-5

**Published:** 2024-02-05

**Authors:** Alessandra Fabi, Luisa Carbognin, Andrea Botticelli, Ida Paris, Paola Fuso, Maria Cristina Savastano, Nicla La Verde, Carla Strina, Rebecca Pedersini, Stefania Guarino, Giuseppe Curigliano, Carmen Criscitiello, Mimma Raffaele, Alessandra Beano, Antonio Franco, Maria Rosaria Valerio, Francesco Verderame, Andrea Fontana, Eva Regina Haspinger, Alessia Caldara, Alba Di Leone, Giampaolo Tortora, Diana Giannarelli, Giovanni Scambia

**Affiliations:** 1 Precision Medicine Unit in Senology, Fondazione Policlinico Universitario A. Gemelli IRCCS, Rome, Italy; 2 Division of Gynecology Oncology, Department of Woman and Child Health and Public Health, Fondazione Policlinico Universitario A. Gemelli IRCCS, Rome, Italy; 3 Medical Oncology Unit, La Sapienza, University of Rome, Policlinico Umberto I, Rome, Italy; 4 Medical Oncology Unit, A.O.U. San Giovanni di Dio e Ruggi D’Aragona, Salerno, Italy; 5 Medical Oncology Unit, ASST Fatebenefratelli Sacco PO Luigi Sacco - Polo Universitario, Milan, Italy; 6 Medical Oncology Unit Azienda Socio-Sanitaria Territoriale Cremona, Cremona, Italy; 7 Medical Oncology Unit ASST Spedali Civili, Brescia, Italy; 8 Medical Oncology Unit Santa Maria della Misericordia Hospital, Urbino, Italy; 9 Department of Oncology and Hemato-Oncology, University of Milan, Milan, Italy; 10 Division of Early Drug Development, European Institute of Oncology, IRCCS, Milan, Italy; 11 Presidio Cassia Sant’andrea, Dipartimento Oncologico, Asl Roma1, Rome, Italy; 12 Department of Medical Oncology1, Città della Salute e della Scienza Hospital, Turin, Italy; 13 Breast Unit, Department of Women, Children and Public Health Sciences, Fondazione Policlinico Universitario Agostino Gemelli IRCCS, Rome, Italy; 14 Medical Oncology, Policlinico Universitario P. Giaccone, Palermo, Italy; 15 Medical Oncology, AO Riuniti Villa Sofia, Cervello, Palermo, Italy; 16 Medical Oncology Unit 2, Azienda Ospedaliero-Universitaria Pisana, Pisa, Italy; 17 Azienda Sanitaria dell’Alto Adige – Ospedale di Merano, Merano, Italy; 18 Santa Chiara Hospital, Trento, Italy; 19 Medical Oncology, Fondazione Policlinico Universitario A. Gemelli IRCCS, Rome, Italy; 20 Medical Oncology, Department of Translational Medicine and Surgery, Università Cattolica del Sacro Cuore, Rome, Italy; 21 Epidemiology and Biostatistics Facility, Fondazione Policlinico Universitario A. Gemelli IRCCS, Roma, Italy; 22 Università Cattolica del Sacro Cuore, Rome, Italy

**Keywords:** Breast cancer, Breast cancer

Correction to: *npj Breast Cancer* 10.1038/s41523-023-00579-2, published online 08 September 2023

In this article the author name Paola Fuso was incorrectly written as Paolo Fuso. The original article has been corrected.

