# Supplementary material for: Real-world ANASTASE study of atezolizumab+nab-paclitaxel as first-line treatment of PD-L1-positive metastatic triple-negative breast cancer
Source: NPJ Breast Cancer. 2023 Sep 8;9:73. doi: 10.1038/s41523-023-00579-2 (PMC10491680; doi:10.1038/s41523-023-00579-2)
Supplement: Supplementary file 1 — Supplementary Materials [file 41523_2023_579_MOESM1_ESM.docx]

**Supplementary Material**

**Supplementary Table 1. Baseline characteristics of patients receiving atezolizumab maintenance therapy compared with no maintenance treatment.**

| **Features** | **Atezolizumab maintenance therapy (n=8)** | **No maintenance treatment (n=44)** | **p-value*** |
| --- | --- | --- | --- |
| Age (years), median (IQR) | 60 (49–63) | 56 (47–67) | 0.97 |
| Metastatic sites:  – Single metastatic site  – Multiple met sites | 8  0 | 21  23 | 0.058 |
| Metastases:  – Lung metastases  – Bone metastases  – Liver metastases | 4  0  0 | 21  9  7 | 0.91  0.16  0.23 |
| Disease stage at initial diagnosis:  – I  – II  – III  – IV | 1  3  3  1 | 3  12  19  10 | 0.82 |
| Disease-free interval  – <12 months  – >12 months | 1  6 | 6  27 | 0.81 |

*** chi-square test**

**Supplementary Table 2. Overall response rate (ORR) according to subgroups.**

|  | **n (%)** | **95% CI** | **p-value*** |
| --- | --- | --- | --- |
| All patients | 22 (42.3%) | 28.9–55.7 |  |
| *HER2*:   - 0 (n=40) - Low (n=11) | 18 (45.0%)  4 (36.4%) | 29.6–60.4  7.9–64.8 | 0.61 |
| Disease-free interval   - <12 (n=7) - ≥12 (n=33) | 1 (14.3%)  15 (45.5%) | 0–40.2  28.5–62.4 | 0.13 |
| *BRCA*:   - Wild-type (n=29) - Mutant (n=8) | 11 (37.9%)  3 (37.5%) | 20.3–55.6  4.0–71.4 | 0.98 |
| *De novo* stage IV:   - No (n=41) - Yes (n=11) | 16 (39.0%)  6 (54.5%) | 24.1–54.0  25.1–84.0 | 0.36 |
| Lung metastases:   - No (n=27) - Yes (n=25) | 13 (48.1%)  9 (36.0%) | 29.3–67.0  17.2–54.8 | 0.38 |
| Liver metastases:   - No (n=45) - Yes (n=7) | 19 (42.2%)  3 (42.9%) | 27.8–56.7  6.2–79.5 | 0.97 |
| Bone metastases:   - No (n=43) - Yes (n=9) | 17 (39.5%)  5 (55.6%) | 24.9–54.1  23.1–88.0 | 0.38 |
| Node metastases:   - No (n=23) - Yes (n=29) | 6 (26.1%)  16 (55.2%) | 8.1–44.0  54.1–91.3 | 0.10 |
| Dominant site:   - Liver (n=7) - Lung (n=24) - Bone (n=5) - Soft tissue (n=16) | 3 (42.9%)  9 (37.5%)  4 (80.0%)  6 (37.5%) | 6.2–79.5  18.1–56.9  44.9–100  13.8–61.2 | 0.35 |
| Number of metastatic sites:   - 1 (n=29) - ≥2 (n=23) | 10 (34.5%)  12 (52.2%) | 17.2–51.8  31.8–72.6 | 0.20 |
| Time from recurrence to first line:   - <60 days (n=26) - ≥60 days (n=26) | 11 (42.3%)  11 (42.3%) | 23.3–61.3  23.3–61.3 | 1.00 |
| Ki-67:   - ≤50% (n=24) - >50% (n=25) | 10 (41.7%)  12 (48.0%) | 21.9–61.4  28.4–67.6 | 0.66 |
| Performance status:   - 0–1 (n=48) - 2–unknown (n=4) | 21 (43.7%)  1 (25.0%) | 29.7–57.8  0.0–67.4 | 0.47 |

*** log-rank test**

**Supplementary Table 3. Progression-free survival (PFS) according to subgroups**

|  | **Median PFS (95% CI)** | **p-value*** |
| --- | --- | --- |
| All patients | 6.3 (3.9–8.7) |  |
| *HER2*:   - 0 (n=40) - Low (n=11) | 6.7 (3.5–9.9)  5.5 (1.4–9.5) | 0.87 |
| Disease-free interval:   - <12 (n=7) - ≥12 (n=33) | 2.8 (2.7–2.9)  6.8 (3.8–9.7) | 0.08 |
| *BRCA*:   - Wild-type (n=29) - Mutant (n=8) | 6.3 (4.2–8.5)  5.3 (0–10.8) | 0.69 |
| *De novo* stage IV:   - No (n=41) - Yes (n=11) | 5.7 (3.9–7.5)  11.2 (0–22.5) | 0.23 |
| Lung metastases:   - No (n=27) - Yes (n=25) | 5.3 (2.6–8.1)  7.4 (3.2–11.6) | 0.33 |
| Liver metastases:   - No (n=45) - Yes (n=7) | 6.7 (4.5–8.9)  4.1 (3.1–5.1) | 0.69 |
| Bone metastases:   - No (n=43) - Yes (n=9) | 7.4 (3.5–11.3)  5.3 (5.2–5.5) | 0.23 |
| Node metastases:   - No (n=23) - Yes (n=29) | 5.7 (2.2–9.2)  7.4 (3.5–11.3) | 0.11 |
| Dominant site:   - Liver (n=7) - Lung (n=24) - Bone (n=5) - Soft tissue (n=16) | 4.1 (3.1–5.1)  7.4 (4.2–10.6)  5.3 (4.7–5.9)  3.5 (0.0–8.1) | 0.88 |
| Number of metastatic sites:   - 1 (n=29) - ≥2 (n=23) | 6.3 (0.1–12.6)  6.7 (4.7–8.7) | 0.94 |
| Time from recurrence to first line:   - <60 days (n=26) - ≥60 days (n=26) | 5.3 (1.6–9.1)  7.4 (2.5–12.3) | 0.76 |
| Ki-67:   - ≤50% (n=24) - >50% (n=25) | 8.3 (4.3–12.3)  6.7 (4.9–8.4) | 0.82 |
| Performance status:   - 0–1 (n=48) - 2–unknown (n=4) | 6.3 (4.0–8.7)  4.8 (0.0–10.9) | 0.29 |

*** log-rank test**

**Supplementary Table 4. Time to next treatment or death (TNT–D) according to subgroups**

|  | **Median TNT–D (95% CI)** | **p-value*** |
| --- | --- | --- |
| All patients | 8.1 (5.5–10.7) |  |
| *HER2*:   - 0 (n=40) - Low (n=11) | 8.7 (5.8–11.6)  6.3 (3.6–9.0) | 0.71 |
| Disease-free interval:   - <12 (n=7) - ≥12 (n=33) | 3.8 (3.5–4.1)  9.1 (6.4–11.8) | 0.24 |
| *BRCA*:   - Wild-type (n=29) - Mutant (n=8) | 7.2 (4.0–10.4)  8.1 (5.2–11.0) | 0.60 |
| *De novo* stage IV:   - No (n=41) - Yes (n=11) | 8.3 (5.2–11.4)  8.1 (1.9–14.2) | 0.59 |
| Lung metastases:   - No (n=27) - Yes (n=25) | 6.5 (5.3–7.7)  8.7 (7.1–10.3) | 0.55 |
| Liver metastases:   - No (n=45) - Yes (n=7) | 8.3 (5.9–10.7)  4.8 (4.0–5.6) | 0.80 |
| Bone metastases:   - No (n=43) - Yes (n=9) | 8.3 (5.5–11.1)  6.9 (6.0–7.8) | 0.34 |
| Node metastases:   - No (n=23) - Yes (n=29) | 6.5 (3.1–9.9)  10.0 (7.4–12.6) | 0.18 |
| Dominant site:   - Liver (n=7) - Lung (n=24) - Bone (n=5) - Soft tissue (n=16) | 4.8 (4.0–5.6)  8.3 (6.0–10.6)  6.9 (5.4–8.4)  6.5 (0.0–13.7) | 0.93 |
| Number of metastatic sites:   - 1 (n=29) - ≥2 (n=23) | 6.5 (2.6–10.4)  8.7 (5.3–12.1) | 0.96 |
| Time from recurrence to first line:   - <60 days (n=26) - ≥60 days (n=26) | 7.2 (4.9–9.4)  8.1 (3.5–12.7) | 0.49 |
| Ki-67:   - ≤50% (n=24) - >50% (n=25) | 8.3 (3.0–13.6)  8.1 (5.4–10.8) | 0.40 |
| Performance status:   - 0–1 (n=48) - 2–unknown (n=4) | 7.2 (4.4–10.0)  8.7 (3.8–13.6) | 0.50 |

*** log-rank test**

**Supplementary Table 5. Comparison between outcomes of the patients in Anastase real life and Impassion130 studies.**

TTD: Time to treatment discontinuation**; TNT–D:** Time to next treatment or death; PFS: Progression-free survival.

| **Patient number** | **ANASTASE real life study (n=52)** | **IMPASSION 130 study (n=185)** |
| --- | --- | --- |
| Overall objective response, n (%) [95% CI] | 22 **(42.3)** [28.9–55.7] | 109 (**58.9**) [51.5–66.1] |
| Complete response, n (%) | 3 (5.8) | 19 (10.3) |
| Partial response, n (%) | 19 (36.5) | 90 (48.6) |
| Stable disease, n (%) | 10 (19.2) | 38 (20.5) |
| Progressive disease, n (%) | 16 (30.8) | 31 (16.8) |
| Patients who had missing data or could not be evaluated, n (%) | 4 (7.7) | 7 (3.8) |
| Duration of response (months), median (95% CI) | 12.7 (4.1–21.4) | 8.5 (7.3–9.7) |
| Cycle to best response (months), median (95% CI) | 3.0 (1–7) | NR |
| TTD (months), median (95% CI) | 5.0 (2.8–7.1) | NR |
| TNT–D (months), median (95% CI) | 8.1 (5.5–10.7) | NR |
| PFS (months), median (95% CI) | 6.3 (95% CI 3.9–8.7) | 7.5 (95% CI 6.7–9.2) |

**Supplementary Figure 1. Overall survival of all the population.** At 12 and 24 months 66.3% and 49.1% of patients were alive, respectively.
