# Supplementary material for: Real-world ANASTASE study of atezolizumab+nab-paclitaxel as first-line treatment of PD-L1-positive metastatic triple-negative breast cancer
Source: NPJ Breast Cancer. 2023 Sep 8;9:73. doi: 10.1038/s41523-023-00579-2 (PMC10491680; doi:10.1038/s41523-023-00579-2)
Supplement: Supplementary file 2 — reporting-summary [file 41523_2023_579_MOESM2_ESM.pdf]

nature portfolio

Corresponding author(s): Alessandra Fabi

Last updated by author(s): Apr 25, 2023

Reporting Summary

Nature Portfolio wishes to improve the reproducibility of the work that we publish. This form provides structure for consistency and transparency in reporting. For further information on Nature Portfolio policies, see our Editorial Policies and the Editorial Policy Checklist.

Statistics

For all statistical analyses, confirm that the following items are present in the figure legend, table legend, main text, or Methods section.

n/a	Confirmed	
☐	☒	The exact sample size (*n*) for each experimental group/condition, given as a discrete number and unit of measurement
☐	☒	A statement on whether measurements were taken from distinct samples or whether the same sample was measured repeatedly
☐	☒	The statistical test(s) used AND whether they are one- or two-sided<br>*Only common tests should be described solely by name; describe more complex techniques in the Methods section.*
☐	☒	A description of all covariates tested
☐	☒	A description of any assumptions or corrections, such as tests of normality and adjustment for multiple comparisons
☐	☒	A full description of the statistical parameters including central tendency (e.g. means) or other basic estimates (e.g. regression coefficient) AND variation (e.g. standard deviation) or associated estimates of uncertainty (e.g. confidence intervals)
☐	☒	For null hypothesis testing, the test statistic (e.g. *F*, *t*, *r*) with confidence intervals, effect sizes, degrees of freedom and *P* value noted<br>*Give P values as exact values whenever suitable.*
☒	☐	For Bayesian analysis, information on the choice of priors and Markov chain Monte Carlo settings
☒	☐	For hierarchical and complex designs, identification of the appropriate level for tests and full reporting of outcomes
☒	☐	Estimates of effect sizes (e.g. Cohen's *d*, Pearson's *r*), indicating how they were calculated

Our web collection on statistics for biologists contains articles on many of the points above.

Software and code

Policy information about availability of computer code

Data collection	Demographics, medical history, BC history, and tumor biology were collected within the CUP, active in Italy, from November 2019 to August 2020
Data analysis	IBM SPSS Statistics for Windows v.28.0 (Armonk, NY) was used for analysis.

For manuscripts utilizing custom algorithms or software that are central to the research but not yet described in published literature, software must be made available to editors and reviewers. We strongly encourage code deposition in a community repository (e.g. GitHub). See the Nature Portfolio guidelines for submitting code & software for further information.

Data

Policy information about availability of data

All manuscripts must include a data availability statement. This statement should provide the following information, where applicable:

- Accession codes, unique identifiers, or web links for publicly available datasets
- A description of any restrictions on data availability
- For clinical datasets or third party data, please ensure that the statement adheres to our policy

The datasets generated during and/or analyzed during the current study are available from the corresponding author upon reasonable request.

Research involving human participants, their data, or biological material

Policy information about studies with human participants or human data. See also policy information about sex, gender (identity/presentation), and sexual orientation and race, ethnicity and racism.

Reporting on sex and gender	The study is focused on breast cancer, the incidence of breast cancer on female gender is much higher than male ones; for this reason in this study no male patients were available
Reporting on race, ethnicity, or other socially relevant groupings	The study is completely unbiased with respect to race, etnicity and social grouping
Population characteristics	The study involved PD-L1-positive metastatic or locally advanced TNBC adult patients who completed at least the first cycle of atezolizumab and nab-paclitaxel treatment
Recruitment	The study involved PD-L1-positive metastatic or locally advanced TNBC adult patients who completed at least the first cycle of atezolizumab and nab-paclitaxel treatment within the CUP AL41712 (active from November 2019 to August 2020).
Ethics oversight	The study was conducted within the protocol approved by the ethics committee of Fondazione Policlinico Universitario A. Gemelli of Rome (Italy; protocol number 25493/22).

Note that full information on the approval of the study protocol must also be provided in the manuscript.

Field-specific reporting

Please select the one below that is the best fit for your research. If you are not sure, read the appropriate sections before making your selection.

☒ Life sciences ☐ Behavioural & social sciences ☐ Ecological, evolutionary & environmental sciences

For a reference copy of the document with all sections, see nature.com/documents/nr-reporting-summary-flat.pdf

Life sciences study design

All studies must disclose on these points even when the disclosure is negative.

Sample size	The study involved PD-L1-positive metastatic or locally advanced TNBC adult patients who completed at least the first cycle of atezolizumab and nab-paclitaxel treatment within the CUP AL41712 (active from November 2019 to August 2020).
Data exclusions	No prior chemotherapy, experimental or targeted systemic therapy for mTNBC was allowed.
Replication	Not relevant to our study
Randomization	Not relevant to our study
Blinding	Not relevant to our study

Reporting for specific materials, systems and methods

We require information from authors about some types of materials, experimental systems and methods used in many studies. Here, indicate whether each material, system or method listed is relevant to your study. If you are not sure if a list item applies to your research, read the appropriate section before selecting a response.

Materials & experimental systems

n/a	Involved in the study	
☒	☐	Antibodies
☒	☐	Eukaryotic cell lines
☒	☐	Palaeontology and archaeology
☒	☐	Animals and other organisms
☐	☒	Clinical data
☒	☐	Dual use research of concern
☒	☐	Plants

Methods

n/a	Involved in the study	
☒	☐	ChIP-seq
☒	☐	Flow cytometry
☒	☐	MRI-based neuroimaging

Clinical data

Policy information about clinical studies

All manuscripts should comply with the ICMJE guidelines for publication of clinical research and a completed CONSORT checklist must be included with all submissions.

Clinical trial registration	ClinicalTrials.gov identifier: NCT05609903
Study protocol	ClinicalTrials.gov identifier: NCT05609903
Data collection	ANASTASE study was a retrospective, multicenter, observational trial conducted in 29 Italian oncology centers among adult patients who completed at least the first cycle of atezolizumab and nab-paclitaxel treatment within the CUP AL41712 (active from November 2019 to August 2020).
Outcomes	The primary objectives were to describe the overall population, including patients who completed at least the first cycle of treatment, estimate the time-to-treatment discontinuation (TTD, defined as the time from initiation of therapy to discontinuation of treatment for any reason), the objective response rate (ORR), using RECIST v1.1, the assessment of clinical benefit at 6 and 12 months, and assess the safety-evaluable population (including all patients who received at least one cycle of the study drug).<br>The secondary objectives were to estimate the duration of response (DoR) among patients with an objective response, defined according to the clinical practice, the median PFS, time to next treatment or death (TNT-D, intended as the time to the start of the therapy to the date of next subsequent systemic treatment initiation or death, whichever occurs first), and OS rate, as well as to describe second-line therapy after atezolizumab plus nab-paclitaxel progression. The incidence of adverse events (AEs) suggestive of potential immune-related etiology was also assessed.<br>The primary objectives were to describe the overall population, including patients who completed at least the first cycle of treatment, estimate the time-to-treatment discontinuation (TTD, defined as the time from initiation of therapy to discontinuation of treatment for any reason), the objective response rate (ORR), using RECIST v1.1, the assessment of clinical benefit at 6 and 12 months, and assess the safety-evaluable population (including all patients who received at least one cycle of the study drug).<br>The secondary objectives were to estimate the duration of response (DoR) among patients with an objective response, defined according to the clinical practice, the median PFS, time to next treatment or death (TNT-D, intended as the time to the start of the therapy to the date of next subsequent systemic treatment initiation or death, whichever occurs first), and OS rate, as well as to describe second-line therapy after atezolizumab plus nab-paclitaxel progression. The incidence of adverse events (AEs) suggestive of potential immune-related etiology was also assessed.
